# Laser-Based Polishing of Additively Manufactured PA12 and PAEK Polymer Components Using a Robotic System

**DOI:** 10.3390/polym18091106

**Published:** 2026-04-30

**Authors:** Emrah Uluz, Leander Metz, Lukas Hedwig, Sebastian Bremen

**Affiliations:** 1 Fraunhofer Institute for Laser Technology ILT, Steinbachstraße 15, 52074 Aachen, Germany; 2 Chair for Laser Technology LLT, RWTH Aachen University, Templergraben 55, 52062 Aachen, Germany; 3 Department of Mechanical Engineering and Mechatronics, FH Aachen University of Applied Sciences, Goethestraße 1, 52064 Aachen, Germany

**Keywords:** laser polishing, additive manufacturing, surface roughness, mechanical properties, selective laser sintering, fused deposition modeling, Polyamide 12, Polyaryletherketone

## Abstract

A non-contact laser polishing method for additively manufactured polymer components with complex three-dimensional geometries is presented, employing a 6-axis robotic system. Robot-guided sample orientation, a quasi-top-hat scanning strategy, and closed-loop temperature control are combined to address curved geometries. On Selective Laser Sintering (SLS)-manufactured Polyamide 12 (PA12) tensile samples with three build orientations and two thicknesses, laser polishing yields up to a 15% increase in tensile strength (*Rm*) and a 50% increase in elongation at break (*A*). For 45°-built 5 mm samples, *Rm* increases from 31.53 MPa to 36.33 MPa and *A* from 6.52% to 9.8%, approaching the tensile strength reported for optimally oriented SLS-printed PA12 Smooth samples of the same grade. For convex–concave PA12 demonstrators, areal roughness (*Sa*) on convex surfaces is reduced from 33.6 µm to 2.7 µm (approximately 92%) and the high-pass-filtered micro-roughness (*Sa_HP_*) on concave surfaces by 98.2% to 0.15 µm. For Fused Deposition Modeling (FDM)-printed Polyaryletherketone (PAEK) samples, *Sa* is reduced from 28.35 µm to 4.1 µm and *Sa_HP_* from 15.98 µm to 0.23 µm (98.6%), despite the high melting temperature and anisotropic raster topography. These results demonstrate that robotic laser polishing constitutes a viable post-processing approach for functionally demanding polymer applications.

## 1. Introduction

The advent of additive manufacturing (AM) has transformed the production of complex and customized polymer components, offering high design flexibility and rapid prototyping capabilities. However, a significant limitation of many AM technologies is their relatively poor surface quality, particularly when compared to conventional manufacturing methods such as injection molding. Elevated surface roughness not only compromises the mechanical properties of AM samples but also adversely affects their hygiene, aesthetics, and tactile characteristics. Consequently, enhancing the surface quality of additively manufactured components has become an important research focus across various industries [1,2,3,4].

To reduce surface roughness, a range of established post-processing techniques, including sanding, chemical smoothing, and abrasive blasting, have been extensively investigated. More recently, laser polishing has emerged as a promising non-contact alternative for improving surface quality. Layher et al. [1] demonstrated that laser polishing can be applied to a broad spectrum of polymers. In similar work, Kumbhar et al. [2] and Chen et al. [3] identified and optimized laser polishing parameters for selected additively manufactured polymers, with Chen et al. [3] also investigating the resulting changes in mechanical properties. Braun [4] introduced a quasi-top-hat scanning strategy for laser polishing and conducted additional parameter studies on various polymeric materials. However, each of these studies primarily focused on planar surfaces, which only partially reflect the demands of actual, three-dimensional samples.

Selective Laser Sintering (SLS) and Fused Deposition Modeling (FDM) represent two of the most widely used polymer AM technologies, each associated with characteristic surface textures. SLS produces samples by selectively fusing a polymer powder bed layer by layer using a focused laser beam, resulting in a staircase effect and a granular surface texture governed by the powder particle size and layer thickness [5,6]. FDM deposits a thermoplastic filament through a heated nozzle in a raster pattern, generating periodic inter-track valleys and a pronounced layer structure [7,8].

The mechanism underlying laser polishing of polymers is based on the selective absorption of laser radiation at the material surface. For CO_2_ laser radiation at *λ* = 10.6 µm, absorption in engineering thermoplastics is strong due to the excitation of molecular vibration modes in the polymer backbone. The absorbed energy locally heats the near-surface material to or above its melting temperature, creating a thin viscous melt layer. Surface tension drives the flow of molten material from peaks into adjacent valleys, thereby reducing the surface amplitude. Upon cessation of irradiation, the material solidifies in the smoothed profile. For SLS-manufactured samples, the remelting process additionally eliminates partially sintered powder particles and collapses surface-connected pores, which are primary sources of surface roughness and local stress concentration [4].

The present study addresses a gap in the existing literature. While laser polishing of additively manufactured polymer surfaces has been demonstrated for planar geometries, no prior study has reported the combination of (1) robotically controlled 6-axis sample orientation enabling defined angle-of-incidence control on non-planar surfaces, (2) a quasi-top-hat scanning strategy applied to three-dimensional curved geometries, and (3) the simultaneous investigation of SLS-processed PA12 and FDM-printed Polyaryletherketone (PAEK) within a single unified process framework. In this study, the application of laser polishing is therefore extended to complex, three-dimensional geometries by using a robotic arm to orient the sample such that the laser beam is maintained at an optimal angle of incidence. This approach enables a more homogeneous distribution of laser energy, resulting in a more uniform smoothing effect. By systematically adjusting and optimizing the laser processing parameters, including power, scanning speed, and remelting strategy, significant reductions in surface roughness (*Sa*) compared with the as-printed state are demonstrated. Surface topography measurements are conducted using white-light interferometry to precisely quantify reductions in both macro- and micro-scale roughness. Additionally, the influence of laser polishing on mechanical performance is evaluated by determining the tensile strength (*Rm*) and elongation at break (*A*) of Polyamide 12 (PA12), thereby assessing whether the improved surface finish translates into enhanced mechanical properties.

## 2. Materials and Methods

### 2.1. Experimental Setup for Laser Polishing

The laser polishing process employed in this study utilized a CO_2_ laser source, operating at a wavelength of λ = 10.6 μm and delivering a maximum output power of 100 W in continuous-wave mode. The laser radiation was subsequently deflected into the working area by a high-precision galvanometric scanner (SCANLAB GmbH, Puchheim, Germany) [9], which was optically coupled to a focusing lens with a focal length of 300 mm. To achieve a uniform energy distribution, the laser beam was deliberately defocused to a diameter of 2 mm. A closed-loop temperature control system was implemented, comprising a pyrometer for real-time temperature monitoring and an acousto-optic modulator for precise modulation of the laser power [4].

**Figure 1:**
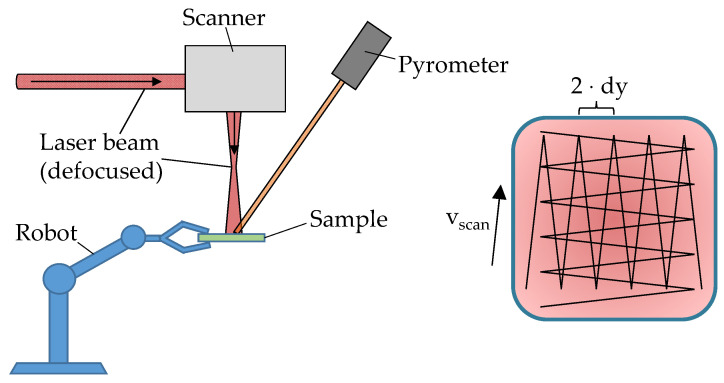
Schematic of the processing setup (**left**) and the laser path (**right**) for the quasi-top-hat scanning strategy.

The robotic system utilized in this work was a 6-axis robotic arm (PROSIX C3 A600S, Epson Deutschland GmbH, Düsseldorf, Germany) [10], which enabled precise orientation and positioning of the sample with respect to the laser beam. The quasi-top-hat scanning strategy, initially developed by Braun [4] was employed to achieve a uniform thermal distribution across the polymer surface (see Figure 1). This scanning approach involved generating scan vectors that followed a meandering pattern, characterized by a track pitch *dy* and a scanning speed *v_Scan_*. Upon completion of each processing pass, the scan vectors were rotated by a predefined angle, and the area was re-illuminated. To ensure a uniform thermal distribution per pass, a random track pitch offset ∆*dy* < *dy* was introduced for each new pass, effectively displacing the scan vectors from their previous positions. The corresponding scanning parameters are summarized in Table 1.

The surface roughness before and after laser polishing was quantitatively assessed using a white-light interferometer (WLI) of type Nexview^TM^ NX2, manufactured by Zygo (Middlefield, CT, USA) [11]. This non-contact optical profiling technique enabled high-resolution topographical analysis of the polished surfaces. The measurement data acquired from the WLI were subsequently analyzed using the proprietary software Mx (version [7.6.0.4], Zygo Corporation, Middlefield, CT, USA), provided by the manufacturer. This software facilitated comprehensive data processing and calculation of relevant surface roughness parameters, allowing for a detailed evaluation of the polishing process’s efficacy. The areal surface roughness parameter *Sa* (ISO 25178-2:2021) was selected as the primary roughness metric over the commonly used profile parameter *Ra* (ISO 4287:1997). For complex three-dimensional and curved geometries, areal parameters provide a statistically more representative characterization, as they evaluate the entire measurement field rather than a single line profile. This is particularly relevant for the convex–concave PA12 demonstrators and the raster topographies of the FDM-printed PAEK sample investigated in this study. Although many published studies report *Ra* values, direct numerical comparison between *Sa* and *Ra* should be made with caution, as the two parameters differ fundamentally in their spatial averaging approach.

### 2.2. Tensile Tests

**Figure 2:**
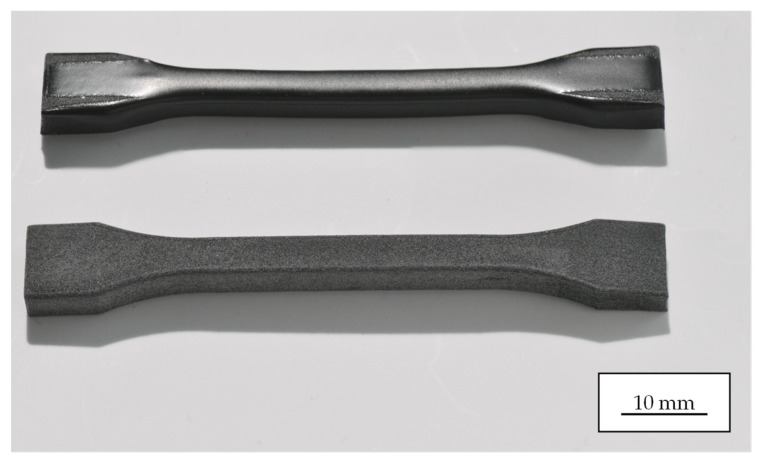
PA12 tensile samples fabricated via SLS printing in the as-printed condition (**bottom**) and after laser polishing (**top**). In the shoulder region of the tensile sample, the laser-polished sample was secured in a dedicated fixture.

Tensile samples (Figure 2) were fabricated via Selective Laser Sintering (SLS) using Sinterit PA12 Smooth powder (Sinterit sp. z o.o. ul. Nad Drwina 10 30-741 Krakow, Poland) [12]. The feedstock, characterized by a melting temperature of 185 °C and a particle size distribution of 19–90 µm [12], contains carbon black additives as indicated by its ‘navy grey’ color, although the specific concentration is not disclosed by the manufacturer. Processing was conducted on a Sinterit Lisa Pro system using parameters (Table 2) consistent with established literature [13,14] to ensure sample reproducibility.

To investigate the mechanical properties, specifically the tensile strength and elongation at break, of the additively manufactured samples, tensile samples were prepared in compliance with the ISO 527-1:2019 and ISO 527-2:2012 standards. The samples were subsequently subjected to tensile testing and evaluated according to standardized procedures, enabling a comparative analysis within each experimental series.

For the purposes of this study, tensile samples were fabricated in three distinct build orientations (horizontal, vertical, and diagonal at 45°) with sample thicknesses of 2 mm and 5 mm. To ensure statistically significant results, five identical samples were prepared for each configuration. Following fabrication, the four lateral surfaces of the tensile samples were laser-polished using the processing parameters listed in Table 3 for PA12. Tensile strength measurements were performed using a Zwick/Roell universal testing machine, model BZ1-MM14450.ZW02 (ZwickRoell GmbH & Co. KG, Ulm, Germany) [15]. The initial preload of 5 MPa was applied at a crosshead speed of 5 mm/min. The tensile modulus was subsequently determined at 1 mm/min, followed by the remaining test at a crosshead speed of 2 mm/min, in accordance with ISO 527-1/-2. Exemplary tensile samples with a sample thickness of 5 mm are shown in Figure 2, illustrating their appearance before and after laser polishing.

### 2.3. Laser Polishing of PA12 Tensile Samples

Prior to laser polishing, the tensile samples underwent a cleaning protocol to remove residual powder particles from the SLS process and sand debris from the sandblasting treatment. This involved a two-step ultrasonic cleaning procedure, in which the samples were immersed in tap water for 10 min at a controlled temperature of 30 °C, followed by a second immersion in distilled water under identical conditions. The cleaned samples were then dried in a thermostatically controlled oven at 120 °C for a minimum of two hours to eliminate any potential effects of moisture absorption on the material’s properties. It should be noted that no control group was included in the present study to independently assess the effect of the cleaning and drying procedure on the mechanical properties of PA12. The drying step was applied to minimize moisture-related effects prior to laser processing. However, a residual influence of this thermal conditioning step on the measured tensile properties cannot be fully excluded. This represents a limitation of the present study and should be addressed in future work through dedicated experiments on samples subjected to the cleaning and drying protocol without subsequent laser polishing.

Dimensional measurements of the tensile specimens before and after laser polishing were not performed as part of this study. Visual inspection (Figure 2) indicated no macroscopic deformation or warping following the polishing process. The consistency of the stress–strain curve shapes before and after polishing (discussed in Section 3.1) further suggests that the effective cross-sectional area in the gauge region was not substantially altered. A precise quantitative assessment of dimensional accuracy is identified as a relevant aspect for future work, particularly for geometrically demanding applications with tight tolerances.

The laser polishing process was conducted using established parameters, based on previous studies on the laser polishing of PA12 [4]. Laser polishing was performed at a setpoint temperature of *T_Set_* = 210 °C and an interaction time of *t_int_* = 240 s. The quasi-top-hat scan strategy was employed, with a defined field size of 20 × 20 mm^2^. Each sample was then translated under temperature-controlled laser radiation at a constant speed, ensuring uniform exposure of all four surfaces (top, bottom, and two side surfaces) to the polishing treatment. The entire laser polishing procedure for a single tensile sample required approximately 60 min.

### 2.4. Laser Polishing of Convex-Concave PA12 Samples

**Figure 3:**
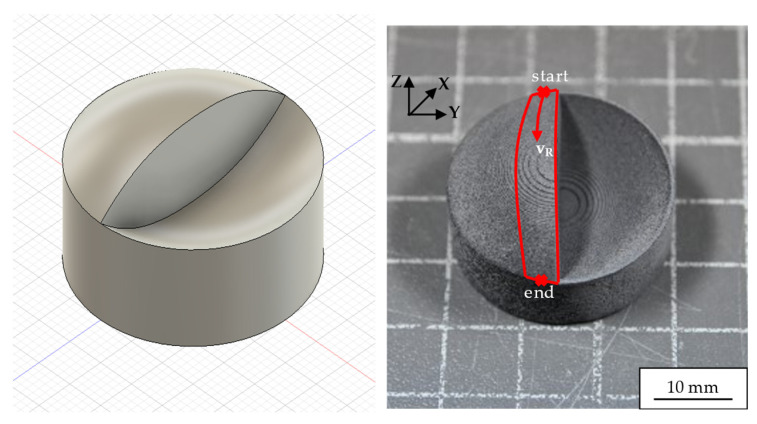
Model of the convex-concave sample (**left**) and the convex-concave sample in its initial state, annotated with the robotic motion path for a laser polishing pass (**right**). Typical surface roughness after a SLS process is *Sa* ≈ 15 µm.

The convex-concave sample underwent the same preprocessing protocol as the tensile samples, involving a two-step ultrasonic cleaning procedure followed by drying in a thermostatically controlled oven. The process parameters for both preprocessing steps remained unchanged. Subsequently, the convex and concave surfaces of the samples were subjected to laser polishing using a three-path strategy. In comparison to the process parameters listed in Table 3, the interaction time tint was reduced and the field size decreased to accommodate the sample geometry. Figure 3 illustrates the trajectory of the three polishing paths, highlighting the sample’s complex motion during processing.

The first path began at a designated starting point, where the sample was tilted by an initial angle using the robot arm. It then underwent continuous rotation under laser radiation, controlled by an EPSON RC+ software script (version [6.2.5], Epson Deutschland GmbH, Düsseldorf, Germany), from the start to the end position. The second path only differed in its start and end coordinates, while following the same fundamental procedure. Conversely, the third path tilted the sample around the X-axis without any rotation around the Y-axis, followed by a linear translation along the X-direction. To ensure complete coverage of unpolished surfaces within a single path, the quasi-top-hat field size was set to 7 × 7 mm^2^. The total duration for all three laser polishing paths was 33 min. Throughout the entire process, both the sample’s motion and laser parameters were carefully controlled to achieve a uniform finish on the complex convex-concave surfaces.

### 2.5. Laser Polishing of PAEK Samples

The PAEK samples were fabricated using FDM from the filament PAEK VICTREX AMTM 200 FIL provided by Victrex plc. (Thornton Cleveleys, UK), which exhibits a melting temperature of 303 °C [16]. The FDM process was extensively demonstrated and validated for additive manufacturing in previous studies [17,18,19]. Building upon these publications, the samples were printed using the processing parameters outlined in Table 4. AM-PAEK materials often exhibit elevated surface roughness due to the layer-based deposition. Consequently, post-processing is typically necessary to enhance surface integrity, mechanical properties, and biocompatibility. Conventional polishing involves both mechanical and chemical approaches, frequently preceded by thermal treatments such as annealing to relieve residual stress and promote crystallinity.

Mechanical polishing can be achieved via sanding with progressively finer abrasives, barrel finishing in rotating drums containing abrasive media (e.g., ceramic or polymer), or ultrasonic polishing for highly intricate geometries. In contrast, chemical polishing utilizes selective dissolution or reflow of the superficial layer, often through acid etching (e.g., sulfuric acid) or vapor smoothing (e.g., acetone) to decrease the overall roughness.

A dedicated polishing step imparts a smoother finish, eliminates micro-cracks, and enhances fatigue resistance. Such refinement is particularly advantageous in medical applications, where diminished surface roughness can improve tissue integration and reduce bacterial adherence. However, conventional polishing poses notable health and safety hazards, mechanical methods risk generating hazardous dust and debris, while chemical methods involve handling corrosive acids and potentially noxious fumes. Elevated processing temperatures may also induce thermal decomposition of PAEK, releasing harmful byproducts. In contrast, the thermal polishing method using laser radiation is implemented within a robotic system. This approach allows fully automated processing in a closed environment equipped with extraction, thereby minimizing any risk to the operator.

Following FDM printing, PAEK typically exhibits an amorphous morphology because the processing temperature remains below its crystallization threshold. While standardized post-processing in an oven induces a crystalline structure on the sample surface, laser polishing offers an alternative, thermally driven approach. Because laser polishing raises the material to its melting point, it both reduces surface roughness and converts the amorphous structure into a crystalline phase.

Unlike the PA12 samples, the PAEK samples were dried in an oven at 150 °C for at least three hours. Braun [4] demonstrated that residual moisture can lead to bubble formation during laser polishing in related materials (e.g., Polyetheretherketone (PEEK)). Since the PAEK samples in this study were manufactured via FDM and were not sandblasted, the ultrasonic cleaning step described in Section 2.3 was omitted.

**Figure 4:**
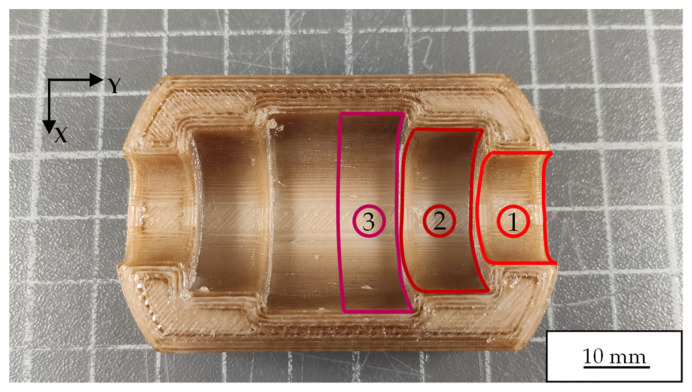
PAEK semi-finished product after FDM printing, showing the marked partial surfaces (1, 2, 3) for laser polishing with synchronized robot arm motion. The numbers indicate the sequential processing. The resulting surface roughness was consistent with that typically achievable through FDM and is in the range of Sa ≈ 25 µm.

Significant height variations among the individual sections of the semi-finished product present a challenge when implementing a uniform processing strategy for the robot arm. To overcome this issue, the partial surfaces defined in Figure 4 were polished sequentially (from 1 to 3) using the quasi-top-hat strategy, ensuring precise and uniform processing. The used laser polishing parameters for PAEK are summarized in Table 5. A process temperature of *T_set_* = 300 °C and an interaction time of *t_int_* = 60 s were employed for each partial surface. From the top view (Figure 4), the polishing process began at the bottom edge of the sample, where the sample was initially tilted around the Y-axis.

During laser exposure, the sample was continuously rotated according to the procedure in Section 2.4, leading to a total processing time of 25 min.

## 3. Results and Discussion

### 3.1. PA12 Tensile Sample

**Figure 5:**
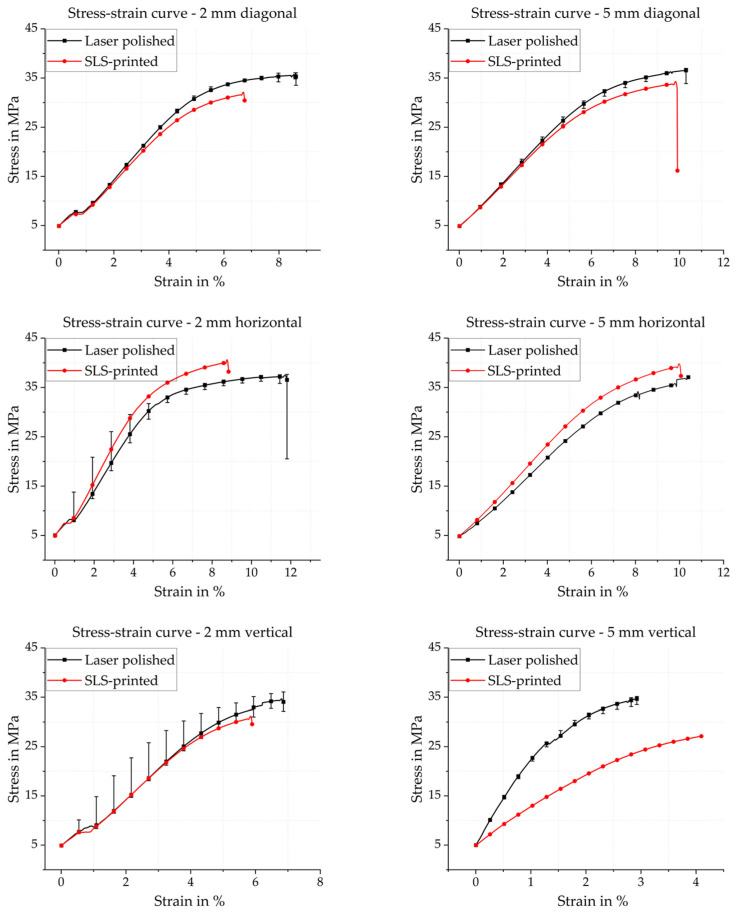
Results of the stress–strain curves of PA12 tensile samples in the as-printed condition (red) and after laser polishing (black) for three build orientations (horizontal, vertical, 45° diagonal) and two sample thicknesses (2 mm and 5 mm).

Following the ISO 527-1/-2 standards described in Section 2.2, tensile tests were conducted on PA12 samples in the as-printed condition and after laser polishing of the four lateral surfaces, as detailed in Section 2.3. All tests were performed with an initial preload of 5 MPa. Figure 5 summarizes the resulting engineering stress–strain curves for all three build orientations (horizontal, vertical, 45° diagonal) and both sample thicknesses (2 mm and 5 mm).

In the as-printed condition, all configurations exhibit a similar qualitative mechanical response. After the initial preload, the engineering stress–strain curves show a linear regime up to tensile stresses of approximately 7.5 MPa, corresponding to predominantly elastic deformation. This is followed by a short transition into the plastic regime and a subsequently nearly linear increase in tensile stress with strain, indicative of strain hardening, until the curves start to level off near the ultimate tensile strength. Failure is characterized by an abrupt drop in tensile stress, which is typical of brittle to moderately ductile fracture in SLS-processed PA12. For the diagonally built samples with a thickness of 5 mm, which are taken as a representative example, the as-printed samples reach an average tensile strength of *Rm* = 31.53 MPa ± 0.90 MPa at a fracture strain of *A* = 6.52% ± 0.44%. After laser polishing, the overall shape of the stress–strain curves remains similar, but the entire curve is shifted towards higher stresses and extended to larger strains (see Figure 5).

The laser-polished samples are again subjected to a preload of 5 MPa, deform elastically up to approximately 7.5 MPa tensile stress, and then transition into the plastic deformation range. Up to a tensile stress of about 27.5 MPa, the curves of the laser-polished tensile samples remain nearly linear with strain, before flattening and approaching the ultimate tensile strength. The laser-polished diagonal samples with 5 mm thickness reach an average tensile strength of *Rm* = 36.33 MPa ± 0.67 MPa and an elongation at break of *A* = 9.8% ± 0.17%, corresponding to an increase of approximately 15% in *Rm* and 50% in *A* relative to the as-printed state. The comparatively small standard deviations indicate good reproducibility of the polishing process.

The trends observed for the diagonal samples are qualitatively consistent across all build orientations and both sample thicknesses, as illustrated in Figure 5. For most combinations of orientation and thickness, the laser-polished samples exhibit higher tensile strengths and larger fracture strains than the corresponding as-printed samples. An exception is observed for horizontally built samples, which already exhibit the highest as-printed tensile strength among all configurations, approaching the reference value reported for optimally oriented SLS-printed samples of the same material grade. For these samples, laser polishing yields a consistent increase in elongation at break but no systematic improvement in *Rm*, which is consistent with a surface-dominated mechanism. The as-printed bulk material is already nearly defect free along the load-bearing direction, and therefore a surface roughness reduction has a proportionally smaller influence on the peak load.

The relative improvement varies between build orientations, reflecting the different roles of layer interfaces and surface defects. In orientations where inter-layer bonding dominates the load transfer (e.g., samples loaded perpendicular to the layer planes), the mechanical response is more sensitive to internal porosity and inter-layer adhesion than to surface condition. In these cases, the relative gain in tensile properties due to surface polishing is less pronounced. In contrast, for orientations with a larger fraction of load-bearing material aligned with the loading direction, the reduction in surface-connected flaws and staircase-induced notches has a stronger influence, leading to a more substantial increase in both tensile strength and elongation at break.

The simultaneous increase in tensile strength and elongation at break suggests that the observed improvements cannot be attributed solely to a geometric effect, such as a slight increase in the effective cross-sectional area due to smoothing of the surface profile. Instead, the combined mechanical and surface characterization presented in Section 2 and Section 3.2 points towards a microstructural mechanism. The controlled remelting of the near-surface region during laser polishing reduces surface-connected pores and sharp notches originating from the SLS process, thereby lowering local stress concentrations. In addition, the quasi-top-hat scanning strategy ensures a relatively homogeneous thermal load, which may promote local healing of inter-particle necks and a more uniform degree of crystallinity near the surface. Both effects increase the effective load-bearing area and delay crack initiation, which is consistent with the enhanced strength and ductility observed in Figure 5 and listed in Table 6.

Overall, the tensile test results demonstrate that the laser polishing strategy developed in this work is compatible with SLS-processed PA12 and does not induce detrimental thermal damage. The absence of detrimental thermal effects is supported by several complementary observations. First, the process temperature setpoint of *T_Set_* = 210 °C is substantially below the thermal degradation onset temperature of PA12. Second, no discoloration, charring, or evolution of decomposition products was observed on any of the processed samples. Third, thermal degradation would be expected to cause embrittlement, manifesting as a reduction in elongation at break and a decline in tensile strength. The experimental results show the opposite trend. Fourth, the overall shape of the stress–strain curves is retained after polishing, with no evidence of an altered failure mode or premature brittle fracture. Taken together, these observations are consistent with a near-surface remelting and smoothing mechanism, and the process is concluded to be compatible with SLS-processed PA12 without inducing detrimental thermal damage.

This finding is particularly relevant for applications where both high surface quality and reliable mechanical performance are required, such as functional prototypes or end-use components with demanding tribological or hygienic requirements. For context, the tensile strength of the laser-polished 45°-built samples with 5 mm thickness (*Rm* = 36.33 MPa ± 0.67 MPa) can be compared to the value of 38.44 MPa reported in the Sinterit PA12 Smooth technical datasheet for samples printed in the X-direction on a Sinterit Lisa X system from fresh powder [12]. It should be noted that this reference value was obtained under different conditions than those used in the present study, namely a different build orientation (X-direction versus 45° diagonal), a fresh powder feedstock (versus the 70:30 used-to-fresh ratio applied here), and a different machine generation. As a consequence, the elongation at break reported in the datasheet for X-direction samples (*A* = 4.55%) [12] is notably lower than the as-printed value measured in the present study (*A* = 6.52% ± 0.44%), which reflects the sensitivity of ductility to build orientation and powder condition in SLS-processed PA12. Despite these differences, the comparison indicates that laser polishing can substantially narrow the mechanical performance gap between differently oriented SLS-processed samples, approaching the values of optimally oriented parts of the same material grade.

The depth of the laser-modified zone was not directly quantified in the present study. Based on the applied processing parameters, a setpoint temperature of *T_Set_* = 210 °C maintained by closed-loop control, a beam diameter of *d_s_* = 2 mm, and a scan speed of *v_Scan_* = 8000 mm/s, thermal penetration is expected to be restricted to the near-surface region as reported by Braun [4], as the controlled surface temperature does not substantially exceed the melting point of PA12. The precise characterization of the modified layer depth, for example by cross-section light microscopy or differential scanning calorimetry (DSC), is identified as a relevant subject for future investigation.

### 3.2. PA12 Convex-Concave Samples

For a more detailed examination of the surface, ten different measurement fields were acquired for each sample using the WLI, distinguishing between the concave and convex surfaces. The 5.5× objective with a 1× zoom level was used for all measurements, providing a suitable compromise between lateral field size and vertical resolution. Following the measurements, the results were analyzed and evaluated using the Mx software (Zygo), which enables consistent computation of both areal roughness Sa and high-pass-filtered micro-roughness (*Sa_HP_*).

**Figure 6:**
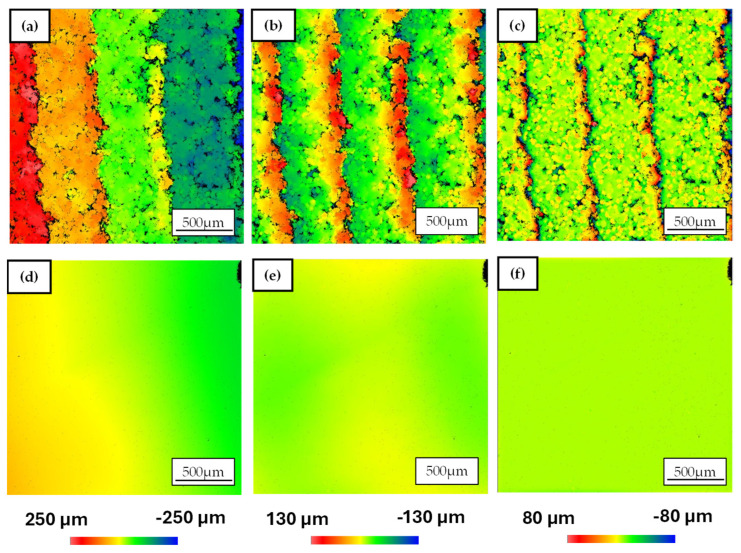
Surface topographies of the convex-concave samples before and after laser polishing: (**a**) initial state, offset-corrected; (**b**) initial state, after subtracting a 2nd-order polynomial; (**c**) initial state, high-pass filtered with *λ_Cut-Off_* = 100 µm; (**d**) laser-polished, offset-corrected; (**e**) laser-polished, after subtracting a 2nd-order polynomial; (**f**) laser-polished, high-pass filtered with *λ_Cut-Off_* = 100 µm.

In Figure 6, the surface topographies of a concave surface are shown for two conditions, the as-fabricated state (AZ) and after laser polishing (LP), using a false-color representation for different evaluation conditions.

In Figure 6a,d, only the offset of the measurement device is subtracted. In Figure 6b,e, a spherical correction is applied by subtracting a second order polynomial to compensate for the global curvature of the convex–concave geometry. Finally, the topography data in Figure 6c,f are filtered with a high pass filter with a cut-off wavelength of *λ_Cut-Off_* = 100 µm in order to isolate the micro-roughness. In the surface topographies shown in Figure 6a, the as-printed condition reveals the characteristic staircase effect of layer wise SLS fabrication, with a step width of approximately 500 µm and a step height of about 100 µm. After applying the spherical correction by subtracting a 2nd-order polynomial (Figure 6b), the underlying curvature is removed and the local deviations due to the SLS process become more prominent. In particular, an accumulation of partially fused powder material is visible in the transition regions between adjacent steps, which can be attributed to local over- or under-exposure during the SLS process and to the discrete layer thickness. The corresponding areal roughness values in the as-printed state are *Sa* = 27.6 µm ± 10.7 µm for concave surfaces and *Sa* = 33.6 µm ± 5.2 µm for convex surfaces, indicating a relatively rough and spatially inhomogeneous surface. The larger standard deviation for the concave regions reflects the stronger local variation in the staircase effect in these geometries. When the high-pass filter is applied (Figure 6c), the long-wavelength curvature and step geometry are largely removed, isolating the micro-roughness generated by the SLS process. It can be observed that relatively homogeneous plateau regions are formed within individual steps, whereas the transition regions between steps cannot be reliably evaluated with this approach due to the strong height gradients. The resulting micro-roughness values are *Sa_HP_* = 8.1 µm ± 2.7 µm for concave and *Sa_HP_* = 10.9 µm ± 2.6 µm for convex surfaces, confirming that the as-printed micro-topography is still relatively coarse, with pronounced asperities at the scale relevant for crack initiation and tribological loading. For reference, the literature reports *Ra* values for SLS-processed PA12 in the as-printed condition typically in the range of 10–30 µm [20], which is broadly consistent with the *Sa* values of 27.6–33.6 µm measured in the present study, given the different spatial averaging inherent to the two parameters.

In Figure 6d, the laser-polished surface is shown with the same offset correction as in Figure 6a. The staircase effect is significantly reduced by laser polishing, and the initially discrete powder particles from the SLS process are melted into a continuous surface layer. The global curvature of the convex–concave geometry is now clearly visible, indicating that the laser treatment has largely eliminated the stepwise topography superimposed on the designed shape. After applying the spherical correction in Figure 6e, the residual surface roughness becomes more apparent. On the surface shown in Figure 6e, shallow depressions with depths of up to approximately 15 µm can be observed. These features are likely associated with localized melt-pool dynamics, for example due to transient overheating, local variations in absorptivity or the collapse of near-surface pores during remelting. Using the same evaluation methodology as in the initial state, the areal roughness of the laser-polished surfaces is determined as *Sa* = 3.8 µm ± 0.8 µm for concave surfaces and *Sa* = 2.7 µm ± 1.9 µm for convex surfaces. Thus, the macroscopic roughness *Sa* is reduced on average by approximately 89% compared to the as-printed condition. To examine the micro-roughness, the surfaces were analyzed using a high-pass filter with a cut-off wavelength (Figure 6f). Compared to the initial state, only isolated small pores and defects remain visible on the laser-polished surface. The micro-roughness *Sa_HP_* is reduced for concave surfaces by 98.2% to *Sa_HP_* = 0.15 µm ± 0.04 µm and for convex surfaces to *Sa_HP_* = 0.66 µm ± 0.43 µm, corresponding to a reduction of 93.9% relative to the as-printed state. A quantitative overview of the roughness parameters and their statistical variation is given in Table 7.

The confidence intervals in Table 7 show no overlap between the as-printed and laser-polished conditions for both *Sa* and *Sa_HP_*, indicating that the observed reductions in roughness are statistically significant for the sample size considered. Moreover, the micro-roughness values achieved after laser polishing (*Sa_HP_* ≤ 1 µm) are within the range typically associated with high-quality technical polymer surfaces and approach the order of magnitude of conventionally manufactured (e.g., injection-molded) PA12 components.

Comparing concave and convex regions, the absolute roughness levels after polishing are slightly lower on the convex surfaces for *Sa*, whereas the concave surfaces exhibit the lowest micro-roughness *Sa_HP_*. This subtle difference likely reflects the interaction between the local angle of incidence of the laser beam, the quasi-top-hat scanning strategy, and heat accumulation on the curved surfaces. Concave regions can retain heat more efficiently during the multiple passes, promoting more complete leveling at the microscale, while convex areas are more efficiently cooled by convection and radiation, which can limit local melt depth. In combination with the mechanical results presented in Section 3.1, the pronounced reduction in surface- and micro-roughness on these convex–concave PA12 samples supports the hypothesis that laser polishing not only smooths the macroscopic staircase geometry but also effectively removes micro-asperities that act as stress concentrators. The resulting reduction in defect population at the surface is consistent with the observed increase in tensile strength and elongation at break and is expected to be beneficial for applications requiring improved fatigue performance, reduced friction, or enhanced cleanability.

### 3.3. Semi-Finished PAEK Samples

**Figure 7:**
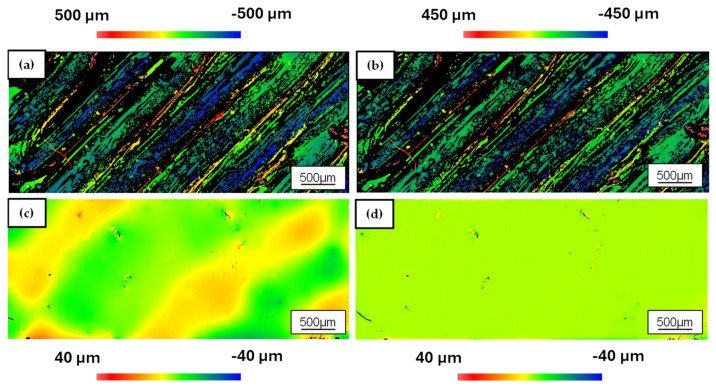
Topographies of the PAEK samples: (**a**) initial state, correction with 4th-order polynomial; (**b**) initial state, high-pass filter with *λ_Cut-Off_* = 100 µm; (**c**) laser-polished, correction with 4th-order polynomial; (**d**) laser-polished, high-pass filter with *λ_Cut_-_Off_* = 100 µm.

For the measurements of the samples in their initial state (AZ) and after laser polishing (LP), the 5.5× objective of the WLI was used. The measurement fields were positioned in the center of partial surface 1 shown in Figure 4, with a measurement area of 5 × 2 mm^2^, in order to capture a representative section of the functional surface while avoiding edge effects. Due to the pronounced curvature and the complex three-dimensional geometry of the semi-finished PAEK samples, mathematical correction using first- or second-order polynomials is not sufficient to obtain a reliable estimate of the overall roughness, as the global shape dominates the measured topography. To approximate the measured surface to a quasi-planar area, a fourth-order polynomial was therefore employed (see Figure 7a,b). This higher-order adjustment permits an adequate description of the complex geometry and suppresses distortions that would arise if lower-order polynomials were used. For Figure 7b,d, the micro-roughness is subsequently analyzed using the previously employed high-pass filter with a cut-off wavelength of *λ_Cut-Off_* = 100 µm, in analogy to the PA12 investigations in Section 3.2.

After correction with the 4th-order polynomial, the diagonally oriented deposition paths of the FDM process become clearly visible in Figure 7a, with a characteristic path width of approximately 500 µm. The grooves between adjacent paths exhibit depths exceeding 500 µm, which is why these areas appear as black regions in Figure 7a,b due to the limited vertical measurement range of the WLI. Consequently, the reported roughness values for the as-printed surfaces should be regarded as conservative estimates. The true peak-to-valley (*PV*) heights are likely even larger. Based on this evaluation, the areal surface roughness in the initial state is *Sa* = 28.35 µm, while the high-pass-filtered micro-roughness is *Sa_HP_* = 15.98 µm. These values confirm that FDM-processed PAEK exhibits a distinctly coarse surface topography, dominated by the raster-wise deposition and the pronounced inter-track valleys. After laser polishing and the associated remelting of the near-surface structures, both the large-scale topographical irregularities and the periodic track spacing introduced by the FDM process are substantially reduced (see Figure 7c).

The previously distinct deposition paths are largely levelled, and the deep inter-track grooves disappear from the measurement window, indicating that the melt has flowed sufficiently to fill the valleys and smooth the ridges. Since the process temperature attained during laser polishing exceeds the glass transition temperature of the material and locally approaches or surpasses the melting temperature, a structural transformation occurs in the PAEK material. The initially amorphous to partially crystalline PAEK near the surface evolves into a predominantly crystalline phase during laser polishing, as discussed in Section 2.5. This thermally induced recrystallization is expected to increase the local stiffness and chemical resistance of the surface layer, in addition to the purely geometric smoothing effect. Quantitatively, the areal surface roughness is reduced from *Sa* = 28.35 µm to *Sa* = 4.1 µm after laser polishing, corresponding to a decrease of approximately 85%. The micro-roughness *Sa_HP_* is lowered from 15.98 µm to 0.23 µm, i.e., by about 98.6% (see Figure 7d). Table 8 summarizes the roughness parameters of the PAEK semi-finished products before and after laser polishing.

Although no standard deviations are available for these measurements, the reduction factors for both *Sa* and *Sa_HP_* are sufficiently large that the effect is clearly technologically relevant. In particular, the micro-roughness level after laser polishing (*Sa_HP_* ≈ 0.2 µm) approaches the regime typically associated with finely polished high-performance polymers and is comparable to or better than many conventionally machined PAEK surfaces.

A qualitative comparison of the false-color maps in Figure 7 further supports this conclusion. In the initial state, the surface is dominated by steep, diagonally oriented ridges and deep valleys, which represent potential initiation sites for cracks and stress corrosion as well as areas of increased bacterial adhesion in medical applications. After laser polishing, the color scale is significantly compressed, and the surface appears nearly uniform at the scale of the measurement, with only isolated small depressions remaining.

In contrast to the SLS-fabricated PA12 samples discussed in Section 3.2, PAEK represents a substantially greater challenge for homogeneous thermal smoothing due to its higher melting temperature and the path-specific geometry inherent to the FDM process. The pronounced reduction in roughness achieved on a complex three-dimensional geometry demonstrates that the developed quasi-top-hat scanning strategy, in combination with robot-assisted samples orientation, is capable of delivering sufficient energy in a spatially uniform manner without distorting the overall shape.

Although a direct experimental comparison with conventional polishing methods was not conducted within the scope of this study, a literature-based assessment provides relevant context. For SLS-processed PA12, mechanical post-processing methods such as barrel finishing and vibratory grinding have been reported to reduce surface roughness to *Ra* values of approximately 12–15 µm [4], and other post processing surface treatments typically achieves Ra values in the range of 2–12 µm for SLS samples [21]. In the present study, laser polishing reduced the areal macro-roughness *Sa* to 2.7–3.8 µm for PA12 convex–concave surfaces and the micro-roughness *Sa_HP_* to values below 0.7 µm, levels that are comparable to or below those achieved by the mechanical methods cited, while requiring no abrasive contact and being applicable to curved geometries without fixturing. For PAEK, conventional mechanical polishing is substantially more demanding due to its high stiffness and chemical resistance, and chemical methods are generally inapplicable owing to PAEK’s excellent resistance to most solvents and acids. The *Sa_HP_* value of 0.23 µm achieved by laser polishing is consistent with values reported for precision-machined and hand polished PAEK surfaces [22]. The non-contact nature of laser polishing, no particle dust and its compatibility with complex three-dimensional geometries represent distinct practical advantages over conventional approaches.

In conjunction with the results presented in Section 3.1 and Section 3.2, these findings indicate that the proposed laser polishing process is suitable not only for SLS-processed PA12 but also for FDM-printed PAEK. The method enables a significant smoothing of complex additively manufactured polymer surfaces and thus has the potential to enhance their functional performance, for example with respect to fatigue resistance, wear behavior, or biocompatibility.

## 4. Conclusions

In this work, the transfer of the laser polishing process to complex three-dimensional polymer surfaces using a robotic arm has been successfully demonstrated. For SLS-processed PA12 convex–concave demonstrators, the areal roughness *Sa* was reduced by approximately 89% on average and the high-pass-filtered micro-roughness *Sa_HP_* was reduced by up to 98.2% to *Sa_HP_* = 0.15 µm. For FDM-printed PAEK samples, *Sa* was reduced from 28.35 µm to 4.1 µm (85%) and *Sa_HP_* from 15.98 µm to 0.23 µm (98.6%). These values approach the roughness level of finely polished technical polymer surfaces. The quasi-top-hat scanning strategy, combined with robot-assisted sample orientation, proved effective even on strongly curved and complex three-dimensional geometries. Furthermore, laser polishing of PA12 tensile samples with 45° diagonal build orientation and 5 mm thickness yielded an increase in tensile strength to *Rm* = 36.33 MPa (from 31.53 MPa, +15%) and an increase in elongation at break to *A* = 9.8% (from 6.52%, +50%), as shown in Figure 5, indicating a simultaneous improvement in strength and ductility without evidence of thermal degradation (see Section 3.1).

The developed process is of particular relevance for several functionally demanding application domains. In the medical field, the achieved micro-roughness levels (*Sa_HP_* ≤ 0.23 µm) are within the range associated with reduced bacterial adhesion and improved tissue integration, which is relevant for polymer implants and surgical instruments. For tribological applications such as gears, cams, and bearing elements, the reduction in micro-roughness is expected to lower friction coefficients and wear rates under contact loading. In the food processing and pharmaceutical sectors, smooth surfaces on complex AM components facilitate cleaning and sterilization, supporting compliance with hygiene regulations. The laser polishing process is particularly attractive for PAEK, whose high stiffness, toughness, and chemical resistance make conventional mechanical and chemical polishing significantly more challenging.

Compared to optimally oriented SLS-printed PA12 Smooth samples of the same material grade (*Rm* = 38.44 MPa in the X-direction, as reported in the manufacturer datasheet and discussed in Section 3.1), the tensile strength achieved for laser-polished 45°-built samples (*Rm* = 36.33 MPa) approaches this reference value, while the process retains the inherent geometric design freedom of additive manufacturing.

Further work is required to realize the full potential of the developed process. In the short term, the characterization of the laser-modified zone depth through cross-section microscopy or differential scanning calorimetry, the quantification of dimensional changes after laser polishing, and the investigation of potential moisture-conditioning effects on PA12 tensile properties should be addressed, as these aspects were identified as limitations of the present study. In the medium term, the development of automated path-planning strategies for arbitrary freeform surfaces, the extension of the process to additional high-performance polymer grades, and the integration of in situ quality monitoring represent key steps towards industrial implementation. In the long term, dedicated validation studies for specific application domains, including medical implants and orthopedic devices, aerospace structural components, and high-cycle tribological systems, are required to demonstrate the combined benefits of improved surface quality and modified near-surface microstructure under service conditions.

## Figures and Tables

**Table 1:** Parameters for Quasi-Top-Hat scanning strategy.

Parameter	Value
Scan speed *v_Scan_*	8000 mm/s
Track pitch *dy*	150 µm
Track pitch offset Δ*dy*	0 µm < Δ*dy* < 150 µm

**Table 2:** SLS processing parameters for manufacturing PA12 samples.

Parameter	Value
Material	PA12 Smooth
Mixed Powder (used:fresh)	70:30
Layer height *h*	0.10 mm
Surface temperature *T_S_*	179 °C
Laser power *P_L_*	5 W

**Table 3:** Process parameters for laser polishing with quasi-top-hat strategy of PA12 tensile samples.

Parameter	Value
Process temperature *T_Set_*	210 °C
Interaction time *t_int_*	240 s
Laser power *P_L_*	70 W
Beam diameter *d_s_*	2 mm
Field size	20 × 20 mm^2^

**Table 4:** Process parameters for FDM of PAEK samples.

Parameter	Value
Material	VICTREX AM 200 FIL
Layer height	0.20 mm
Nozzle temperature	390 °C
Bed temperature	150 °C
Printing speed	50 mm/s
Nozzle diameter	0.4 mm

**Table 5:** Process parameters for laser polishing with quasi-top-hat strategy of PAEK samples.

Parameter	Value
Temperature set point	300 °C
Interaction time	60 s
Laser power	50 W
Beam diameter	2 mm
Scanning strategy	Quasi-Top-Hat
Scan field size	8 × 8 mm^2^–10 × 10 mm^2^

**Table 6:** Tensile strength (*Rm*) and elongation at break (*A*) of PA12 specimens for three build orientations and two specimen thicknesses, in the as-fabricated state (AZ) and after laser polishing (LP). Values are reported as the mean ± standard deviation (SD). The number of samples evaluated per configuration (*n* = 5).

Build Orientation	Thickness in mm	Condition	*Rm* [MPa] (Mean ± SD)	*A* [%] (Mean ± SD)
45°	2	Before LP	31.1 ± 0.50	4.93 ± 0.53
45°	2	After LP	35.67 ± 0.53	7.14 ± 0.51
Horizontal	2	Before LP	40.04 ± 0.41	4.19 ± 0.31
Horizontal	2	After LP	36.81 ± 1.86	4.90 ± 0.55
Vertical	2	Before LP	30.15 ± 2.08	7.54 ± 0.51
Vertical	2	After LP	33.7 ± 2.15	9.12 ± 0.50
45°	5	Before LP	31.53 ± 0.90	6.52 ± 0.44
45°	5	After LP	36.39 ± 0.67	9.80 ± 0.17
Horizontal	5	Before LP	38.89 ± 0.97	3.46 ± 0.39
Horizontal	5	After LP	36.63 ± 1.88	7.41 ± 0.57
Vertical	5	Before LP	27.08 ± 1.62	9.01 ± 1.03
Vertical	5	After LP	34.37 ± 0.84	8.95 ± 1.34

**Table 7:** Surface roughness of PA12 convex–concave samples before and after laser polishing (LP). Ten measurement fields were evaluated for each surface type and condition (*n* = 10), and 95% confidence intervals (CI) were calculated.

Surface Type	Condition	*Sa* [µm]	95% CI for *Sa* [µm]	*Sa_HP_* [µm]	95% CI for *Sa_HP_* [µm]
Concave	Initial state	27.6 ± 10.7	19.9–35.3	8.1 ± 2.7	6.2–10.0
Concave	LP	3.8 ± 0.8	3.2–4.4	0.15 ± 0.04	0.12–0.18
Convex	Initial state	33.6 ± 5.2	29.9–37.3	10.9 ± 2.6	9.0–12.8
Convex	LP	2.7 ± 1.9	1.3–4.1	0.66 ± 0.43	0.35–0.97

**Table 8:** Surface roughness of PAEK semi-finished products before (AZ) and after laser polishing (LP).

Condition	*Sa* [µm]	*Sa_HP_* [µm]
Initial state	28.35	15.98
LP	4.1	0.23

## Data Availability

The original contributions presented in this study are included in the article. Further inquiries can be directed to the corresponding authors.
